# The motor cortex: a network tuned to 7-14 Hz

**DOI:** 10.3389/fncir.2013.00021

**Published:** 2013-02-21

**Authors:** Manuel A. Castro-Alamancos

**Affiliations:** Department of Neurobiology and Anatomy, Drexel University College of MedicinePhiladelphia, PA, USA

**Keywords:** oscillatory activity, motor cortex, behavioral state, potassium channels, voltage-gated, excitatory synapses

## Abstract

The neocortex or six layer cortex consists of at least 52 cytoarchitectonically distinct areas in humans, and similar areas can be distinguished in rodents. Each of these areas has a defining set of extrinsic connections, identifiable functional roles, a distinct laminar arrangement, etc. Thus, neocortex is extensively subdivided into areas of anatomical and functional specialization, but less is known about the specialization of cellular and network physiology across areas. The motor cortex appears to have a distinct propensity to oscillate in the 7–14 Hz frequency range. Augmenting responses, normal mu and beta oscillations, and abnormal oscillations or after discharges caused by enhancing excitation or suppressing inhibition are all expressed around this frequency range. The substrate for this activity may be an excitatory network that is unique to the motor cortex or that is more strongly suppressed in other areas, such as somatosensory cortex. Interestingly, augmenting responses are dependent on behavioral state. They are abolished during behavioral arousal. Here, I briefly review this evidence.

## MOTOR CORTEX IS MORPHOLOGICALLY DISTINCT

The location, general organization, and cytoarchitectural correlates of the principal fields of the rat somatosensory and motor cortex have been established with anatomical and electrophysiological techniques. The primary motor cortex, identified as the region of cortex where intracortical stimulation produces movements at the lowest thresholds, occupies two cytoarchitectonic fields. The majority of motor cortex coincides with a distinct frontal agranular area called the *lateral agranular* field that represents the head including the whiskers, the trunk, and part of the forelimb (Hall and Lindholm, 1974; Donoghue and Wise, 1982). The remainder of motor cortex is contained in the rostral part of the granular parietal cortex. This part of the primary motor cortex overlaps with the somatosensory cortex and contains the representation of the hindlimb and part of the forelimb. The primary somatosensory cortex lies within the parietal cortex and has two subdivisions. One subdivision contains the representation of cutaneous receptors and coincides with cytoarchitectonically distinct areas that are marked by their densely *granular* layer IV. Each of these granule cell aggregates contains the representation of cutaneous inputs from a particular region of the body such as the forelimb, hindlimb, and whiskers (barrel cortex). Surrounding the granular fields is a second subdivision termed the *dysgranular* field, which has a thin layer IV and cells respond poorly to light tactile stimuli (Welker, 1971, 1976).

Despite the efforts to conceive a canonical cortical circuit (Douglas et al., 1995), the reality is that neocortical areas are morphologically, physiologically, and consequently functionally distinct (Brodmann, 1905; Zilles, 1985; Creutzfeldt and Creutzfeldt, 1993; Mountcastle, 1998). At the laminar, cellular, and synaptic level the motor (mostly agranular) and somatosensory (mostly granular) areas are quite different. In particular, the motor cortex contains a very large layer V and an almost inexistent layer IV, while the opposite is the case for the somatosensory cortex. Most if not all the excitatory cells in motor cortex are pyramidal cells, and the largest of these are encountered in layer V with apical dendrites that reach into layer I (DeFelipe and Farinas, 1992). The cortex also contains a variety of GABAergic non-pyramidal cells (Kawaguchi and Kubota, 1997; Somogyi et al., 1998). The large extension of layer V in motor cortex suggests that it may contain cell populations absent in somatosensory cortex. Indeed, the motor cortex contains a unique layer V cell population, called Betz cells or giant pyramidal cells (Betz, 1874). Interestingly, these cells become increasingly larger in more evolved species (Sherwood et al., 2003). They are generally encountered in layer Vb and can be up to 20 times larger than other pyramidal cells in the same layer. These obvious morphological differences, and many others in more subtle connectivity, are likely to contribute to the distinct specialization of cellular physiology across areas. Here I focus on one such physiological specialization of the motor cortex, its distinct tuning to the 7–14 Hz frequency band.

## 7–14 Hz AUGMENTING RESPONSES IN MOTOR CORTEX

**FIGURE 1:**
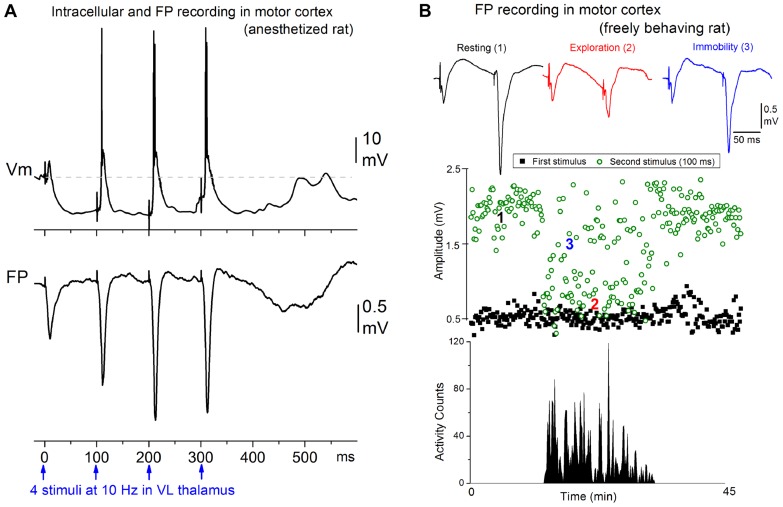
**Augmenting responses in motor cortex driven by ventrolateral (VL) thalamus activity at 7–14 Hz**. **(A)** Intracellular (layer V) and field potential (FP) recordings showing an augmenting response evoked in motor cortex by electrical stimulation in VL at 10 Hz in a ketamine anesthetized rat (Castro-Alamancos and Connors, 1996b). **(B)** Behavioral state dependency of augmenting responses in a freely behaving rat (Castro-Alamancos and Connors, 1996c). The animal was allowed to freely explore the cage and locomotor activity was monitored with photobeam detectors. The amplitude of the responses to the first and second stimulus delivered at 10 Hz is plotted. The augmenting response is induced during periods of resting and awake immobility but is inactivated during periods of active exploration.

Afferent activity between 7 and 14 Hz triggers augmenting responses in the motor cortex. Thus, progressively augmenting excitatory responses are generated in the motor cortex when the ventrolateral (VL) nucleus of the thalamus is stimulated electrically at 7–14 Hz, but only decrementing responses are observed in the primary sensory cortex when the ventroposterior medial (VPM) nucleus of the thalamus is stimulated (Castro-Alamancos and Connors, 1996b,c,d). The spatiotemporal response patterns can be revealed by measuring extracellular voltage at regular intervals through the cortical depth, and calculating a map of current source density (CSD). Electrical stimulation delivered in VL produces a response in motor cortex consisting of a relatively small current sink in layer V and a corresponding current source in upper layers (Castro-Alamancos and Connors, 1996d), which is consistent with the anatomical projections of this thalamic nucleus (Herkenham, 1980; Castro-Alamancos and Connors, 1997). Repetitive electrical stimulation between 7 and 14 Hz generates a strong enhancement of the layer V current sink, and related excitatory postsynaptic potentials recorded intracellularly. These augmenting responses can be generated only within a narrow time interval following a conditioning stimulus; the second stimulus must occur between 50 and 200 ms. The intracellular correlate of the narrow time interval for generating the augmenting response is a prominent hyperpolarization of layer V cells generated by inhibitory postsynaptic potentials recruited through VL thalamocortical fibers stimulation of inhibitory interneurons (**Figure 1A**). The termination of the augmenting interval is marked by a long-latency event that peaks at around 200 ms, and is evident as a slow, negative field potential rebound component that corresponds to a current sink in layer V and a broad depolarizing event intracellularly; akin to a short Up state. Although several cellular mechanisms have been proposed to explain augmenting responses, the underlying mechanisms are not completely established.

Interestingly, the strength of augmenting is dependent on behavioral state (**Figure 1B**). During anesthesia, slow-wave sleep, and quiet periods of awake immobility augmenting is maximal, but as soon as the subject switches to a more activated state, such as exploration or skilled motor behavior (reaching and grasping), the augmenting response is abolished (Castro-Alamancos and Connors, 1996c). During the generation of augmenting responses, activity becomes synchronized over large regions of neocortex (Castro-Alamancos and Connors, 1996b,d). We found that a single VL stimulus, which does not elicit augmenting, activates a very limited region of motor cortex, but a second VL stimulus arriving within 50–200 ms, which elicits augmenting, synchronized the activity across an area several times larger in motor cortex. Thus, augmenting reflects a powerful mechanism that can dynamically change, depending on behavioral state, the level of synchronization of activity in large regions of neocortex. Highly synchronous activity throughout neocortex may impede normal information processing because cortical neurons will all respond in the same fashion at the same time, degrading spatial resolution.

## 7–14 Hz NETWORK OSCILLATIONS IN MOTOR CORTEX

**FIGURE 2:**
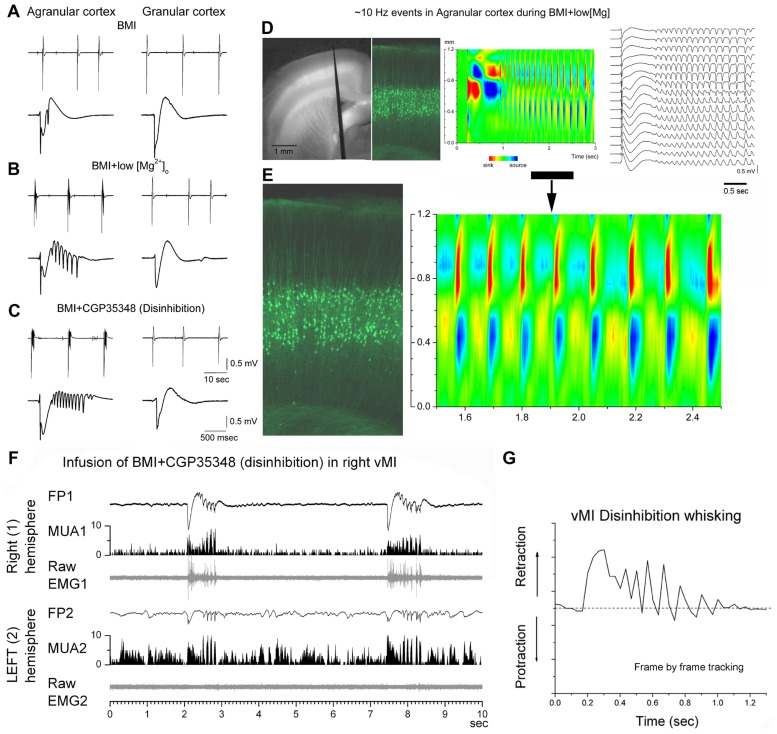
**7–14 Hz oscillations are caused by low [Mg^2+^]_o_ buffer or disinhibition in the motor cortex but not in the somatosensory cortex**. **(A–C)** Field potential (FP) recordings in neocortical slices showing typical spontaneous events recorded in motor (agranular) cortex (left panels) and in somatosensory (granular) cortex (right panels) during block of GABA_A_ receptors with bicuculline (BMI) **(A)**, subsequent lowering of [Mg^2^^+^]_o_ **(B)**, or subsequent block of GABA_B_ receptors **(C)**. Note the occurrence of 7–14 Hz oscillations only in agranular cortex. In each panel, the upper trace has a long time scale and the lower trace shows a close-up of a spontaneous discharge (Castro-Alamancos and Tawara-Hirata, 2007). **(D)** Recording from a fluorescent slice of an Thy1-eYFP mouse using a 16-channel silicon probe. The left panel shows a light/fluorescent image of the probe position in the motor cortex during the experiment. The middle left panel show a fluorescent image taken at the probe location revealing the layer V fluorescent cells. The current source density (CSD) and FP plots show a 7–14 Hz oscillation event. The CSD and fluorescent image are scaled to match the cortical layers location with the location of the current flow (Castro-Alamancos and Tawara-Hirata, 2007). **(E)** Close-up of the 7–14 Hz event shown in **(D)**. **(F)** 7–14 Hz oscillations caused by disinhibition in the whisker motor cortex (vMI) of a freely behaving rat. Bilateral field potential (FP) and multi-unit (MUA) activity recorded from the vMI during disinhibition caused by application of BMI + CGP (0.1 + 2 mM, 0.2 μl volume) into the right vMI. The corresponding raw electromyography (EMG) activity measured from the contralateral whisker pad is also displayed below the FP and MUA. 1 refers to the infused hemisphere and the related (contralateral) whisker pad, 2 refers to the non-infused hemisphere and the related whisker pad (Castro-Alamancos, 2006). **(G)** 7–14 Hz oscillations of vMI caused by disinhibition produce cycles of vibrissa retractions. Frame by frame tracking of a vibrissa movement caused by vMI disinhibition. Units in the *y*-axis are relative pixels (see Castro-Alamancos, 2006).

Enhancing excitation or dampening inhibition leads to 7–14 Hz network oscillations in motor cortex both in slices and *in vivo* (**Figures 2A–C**). Disinhibition (block of GABA_A_ and GABA_B_ receptors) in motor cortex, but not in somatosensory cortex, produces highly synchronous oscillations or afterdischarges at 7–14 Hz (Castro-Alamancos, 2000; Castro-Alamancos and Rigas, 2002). Low [Mg^2^^+^]_o_ buffers (~0.1 mM) that enhance network excitability, also produce 7–14 Hz oscillations in slices (Silva et al., 1991; Flint and Connors, 1996; Castro-Alamancos et al., 2007), which occur more readily in motor cortex than in somatosensory cortex (Castro-Alamancos and Tawara-Hirata, 2007). However, 7–14 Hz oscillations can be unmasked in somatosensory cortex by suppressing specific outward currents, as described below (Castro-Alamancos and Tawara-Hirata, 2007). These results suggest that specific outward currents, such as the slowly inactivating K^+^ current (*I*_D_), may inhibit the ability of somatosensory cortex to generate 7–14 Hz oscillations.

## WHAT IS THE SUBSTRATE OF 7–14 Hz RESONANCE IN MOTOR CORTEX?

When thinking about the mechanisms responsible for 7–14 Hz resonance in motor cortex it is important to consider the following. First, a pure excitatory network of interconnected pyramidal cells generates 7–14 Hz oscillations in motor cortex because these oscillations are present in the absence of GABAergic transmission (Castro-Alamancos and Borrell, 1995; Flint and Connors, 1996; Castro-Alamancos, 2000; Castro-Alamancos and Rigas, 2002). In fact, GABA_B_ receptors normally suppress the generation of 7–14 Hz oscillations because when GABA_B_ receptors are blocked these oscillations are readily expressed, as long as there is a robust excitatory drive caused by GABA_A_ receptor block (Castro-Alamancos, 2000; Castro-Alamancos and Rigas, 2002). This implies that blocking GABA_B_ receptors may unmask the mechanism(s) responsible for generating 7–14 Hz oscillations in the motor cortex.

Second, selective alpha-amino-3-hydroxy-5-methyl-4-isoxazolepropionic acid (AMPA) receptor antagonists completely abolish 7–14 Hz oscillations with little effects on the discharges that trigger them (Castro-Alamancos and Rigas, 2002; Castro-Alamancos et al., 2007). This indicates that fast synaptic excitation is critical for generating and/or synchronizing 7–14 Hz oscillations. In fact, slower *N*-methyl-D-aspartic acid (NMDA)-mediated synaptic currents cannot sustain 7–14 Hz oscillations without AMPA currents. NMDA receptor antagonists reduce the power and enhance the frequency of the oscillations but do not abolish them (Castro-Alamancos and Rigas, 2002; Castro-Alamancos et al., 2007). Therefore, slow NMDA-mediated synaptic currents modulate but are not required for generating 7–14 Hz oscillations.

Third, drugs that suppress the low-threshold calcium current (*I*_T_) and the hyperpolarization-activated cation current (*I*_H_) do not suppress 7–14 Hz oscillations in motor cortex (Castro-Alamancos et al., 2007). Thus, *I*_T_ and *I*_H_ are not required for 7–14 Hz oscillations.

Fourth, it has been known for some time that in addition to the transient Na^+^ current, cortical pyramidal neurons express a persistent Na^+^ current, *I*_Nap_, which appears as a subthreshold inward rectification (Hotson et al., 1979; Connors et al., 1982; Stafstrom et al., 1982, 1984, 1985). Several drugs have been shown to somewhat selectively suppress the persistent component of the Na^+^ current with less effect on the transient component, such as phenytoin (Quandt, 1988; Segal and Douglas, 1997) and riluzole (Urbani and Belluzzi, 2000; Kononenko et al., 2004; Niespodziany et al., 2004). These drugs, known to suppress *I*_Nap_, abolish 7–14 Hz oscillations at doses that have little effects on synaptic transmission (Castro-Alamancos et al., 2007). Thus, *I*_Nap_ appears to be critical for 7–14 Hz oscillations.

Fifth, blockers of voltage-dependent K^+^ channels have significant effects on 7–14 Hz oscillations of motor cortex. Pyramidal cells express a voltage-dependent K^+^ current, the M-current (*I*_M_), which activates positive to -60 mV and does not inactivate (Halliwell and Adams, 1982; Storm, 1990). *I*_M_ is blocked by XE991 and linopirdine in pyramidal cells (Aiken et al., 1995; Hu et al., 2002; Yue and Yaari, 2004). *I*_M_ blockers abolish 7–14 Hz oscillations in motor cortex (Castro-Alamancos et al., 2007). Thus, *I*_M_ appears to be critical for 7–14 Hz oscillations.

Sixth, CSD analysis shows that current flow in motor cortex during 7–14 Hz oscillations is strongest in layers V and II–III, and appears to propagate between the soma and apical dendrites of layer V cells (Castro-Alamancos and Rigas, 2002; see **Figures 2D,E**). This suggests a critical role for layer V cells in the generation of 7–14 Hz oscillations in motor cortex.

These results indicate that a network of pyramidal cells (interconnected by fast AMPA-mediated glutamatergic synapses) that intrinsically express *I*_Nap_ and *I*_M_ are involved in the generation of 7–14 Hz oscillations. Possibly, 7–14 Hz oscillations of motor cortex involve the interplay between synaptic (AMPA) and intrinsic inward (*I*_Nap_) and outward currents (*I*_M_ and perhaps other non-inactivating voltage-dependent K^+^ currents) in layer V pyramidal cells. Interestingly, such a scheme is supported by computational modeling (Golomb et al., 2006).

## MOTOR CORTEX vs. SOMATOSENSORY CORTEX

An important question is, why are 7–14 Hz oscillations generated in motor but not in somatosensory cortex (i.e., during disinhibition or low [Mg^2^^+^]_o_)? There are at least two main possibilities to explain this result. The somatosensory cortex may be incapable of generating 7–14 Hz oscillations because it lacks the necessary intrinsic or synaptic mechanisms. Alternatively, some current(s) inhibits the ability of somatosensory cortex to generate 7–14 Hz oscillations. If the later is the case, blocking the “inhibiting current” should unmask 7–14 Hz oscillations. But what current may be responsible for keeping a pure excitatory network in somatosensory cortex from generating 7–14 Hz oscillations? In the absence of GABAergic inhibition, intrinsic K^+^ currents are the counterbalance of excitation. Hence, it is possible that K^+^ currents in somatosensory cortex impede the generation of 7–14 Hz oscillations (just like GABA_B_ receptors suppress 7–14 Hz oscillations in motor cortex). CA1 and layer V pyramidal cells express three major types of voltage-dependent K^+^ currents in the soma and dendrites (Storm, 1988, 1990; Hoffman et al., 1997; Bekkers, 2000a,b; Korngreen and Sakmann, 2000; Bekkers and Delaney, 2001); a transient current that rapidly activates and inactivates (*I*_A_), a more slowly inactivating current (*I*_D_), and a sustained delayed rectifier (*I*_K_). Importantly, these three voltage-dependent K^+^ current components have well-known sensitivities to K^+^ channel blockers; low doses of 4-aminopyridine (4-AP; μM range) block the slowly inactivating K^+^ current *I*_D_, with little effect on *I*_K_ and *I*_A_. Whereas, tetraethylammonium (TEA) at high doses (10–30 mM) blocks the sustained delayed rectifier *I*_K_ plus other K^+^ currents (Storm, 1988, 1990; Hoffman et al., 1997; Bekkers and Delaney, 2001). Interestingly, 7–14 Hz oscillations are unmasked in somatosensory cortex by low doses of 4-AP, which blocks *I*_D_ (Castro-Alamancos and Tawara-Hirata, 2007). These results indicate that specific outward currents, such as the slowly inactivating K^+^ current (*I*_D_), inhibit the ability of somatosensory cortex to generate 7–14 Hz oscillations because when this current is suppressed 7–14 Hz oscillations are expressed in somatosensory cortex. It is worth noting that the substrate unmasked in somatosensory cortex to produce 7–14 Hz oscillations may be different than the one normally engaged in motor cortex.

There are other known physiological differences between motor and somatosensory cortex that may contribute to their different susceptibility to 7–14 Hz oscillations. Pyramidal cells of the neocortex come in two major types: regular spiking and bursting (Connors and Gutnick, 1990). Regular spiking cells can be further differentiated according to the amount of spike-frequency adaptation. Bursting cells present different degrees; from single bursts to repetitive bursting. Studies *in vivo* have shown that the main electrophysiological types described in somatosensory cortex are also found in cat (Baranyi et al., 1993a,b) and rat motor cortex (Pockberger, 1991). Intriguingly, a unique “accelerating” firing type mediated by a Kv1 subunit is found in layer V pyramidal cells of rodent motor cortex but not in somatosensory cortex (Miller et al., 2008).

Few studies have directly compared the synaptic response properties of somatosensory and motor cortex. For example, theta-burst stimulation applied to layer V–IV produces long-term potentiation (LTP) in layer III of somatosensory (granular) cortex, while the same procedure in the adjacent motor (agranular) neocortex is less effective unless inhibition is suppressed (Castro-Alamancos et al., 1995). These differences appear to be related to the different response properties of synaptic pathways in these neocortical areas (Castro-Alamancos and Connors, 1996a). During tetanic stimuli the granular cortex, but not the agranular cortex, generated a strong short-term enhancement that correlated well with the subsequent expression of LTP (Castro-Alamancos and Connors, 1996a). The variation in short-term enhancement was due to pathway-specific differences in the short-term regulation of NMDA receptor-dependent responses, which in turn determine the differences in long-term NMDA receptor-dependent potentiation. Specifically, NMDA receptor-mediated responses are more strongly depressed in agranular than in granular cortex during high-frequency stimulation.

## 7–14 Hz MOTOR CORTEX OSCILLATIONS ARE FUNCTIONALLY RELEVANT

In humans and non-human primates, oscillatory activity in the beta frequency range (14–30 Hz) is commonly observed in sensorimotor cortex in relation to motor behavior (Donoghue et al., 1998; Saleh et al., 2010). Mu waves (12–18 Hz) occur in the sensorimotor cortex of awake cats during behavioral immobility and are blocked by active movement (Bouyer et al., 1983). In rodents, the intact motor cortex produces 7–14 Hz synchronized oscillations during specific types of whisker movements, such as twitching (Semba and Komisaruk, 1984), which may actually be an abnormal behavioral state that occurs in certain rodent strains (Shaw, 2004). Oscillations occur also during or in anticipation of active whisking (Ahrens and Kleinfeld, 2004; Friedman et al., 2006). These oscillations are usually much less widespread and less synchronous than those occurring during abnormal conditions, such as when inhibition is suppressed. Thus, 7–14 Hz oscillations induced in behaving rats by motor cortex disinhibition produce rhythmical jerks and tremor-like phase-locked movements of the contralateral body (Castro-Alamancos, 2006; **Figure 2F**). Whiskers produce abnormal retractions that contrast with the normal protractions during exploratory whisking (Castro-Alamancos, 2006; **Figure 2G**). This abnormal motor behavior resembles a cortical myoclonus in humans with *Epilepsia Partialis Continua*, which is also associated with large amplitude discharges at 7–14 Hz in the frontal lobes (Obeso et al., 1985; Chauvel et al., 1992).

## CONCLUSION

Several lines of evidence presented here support the notion that the motor cortex contains a unique excitatory network that is tuned to the 7–14 Hz frequency band, and this network may be present but more strongly suppressed in somatosensory cortex. Under normal conditions, afferent activity from the VL thalamus at 7–14 Hz drives augmenting responses in the motor cortex. Augmenting responses are very dependent on behavioral state. They are abolished during sensorimotor processing, such as skilled movement or active exploration, but are fully expressed during sow-wave sleep, anesthesia, and awake quiescence. This activity may be linked to the ability of motor cortex to oscillate at mu and beta frequencies during certain states. Finally, the 7–14 Hz activity in motor cortex is fully expressed when inhibition is suppressed or excitation is enhanced, and it can drive abnormal movements that resemble those expressed during partial epilepsies.

## Conflict of Interest Statement

The author declares that the research was conducted in the absence of any commercial or financial relationships that could be construed as a potential conflict of interest.
